# Case report: acute care management of severe opioid withdrawal with IV fentanyl

**DOI:** 10.1186/s13722-022-00305-6

**Published:** 2022-04-05

**Authors:** Pouya Azar, Jean N. Westenberg, Martha J. Ignaszewski, James S. H. Wong, George Isac, Nickie Mathew, R. Michael Krausz

**Affiliations:** 1 Complex Pain and Addiction Service, Vancouver General Hospital, DHCC, Floor 8-2775 Laurel Street, Vancouver, BC V5Z 1M9 Canada; 2 Department of Psychiatry, Faculty of Medicine, University of British Columbia, Vancouver, BC Canada; 3 BC Children’s Hospital, Vancouver, BC Canada; 4 Division of Critical Care Medicine and Department of Anesthesiology, Pharmacology & Therapeutics, Faculty of Medicine, University of British Columbia, Vancouver, BC Canada; 5 BC Mental Health & Substance Use Services, Provincial Health Services Authority, Vancouver, BC Canada

**Keywords:** Fentanyl, Opioid use disorder, Withdrawal management, Pain management, Acute care, Patient centered care, Inpatient

## Abstract

**Background:**

An increasing number of individuals who use drugs in North America are preferentially consuming fentanyl over other opioids. This has significant consequences on the treatment and management of opioid use disorder (OUD) and its concurrent disorders, especially in acute care if opioid requirements are not met.

**Case presentation:**

We present a patient with severe OUD and daily injection of fentanyl, admitted to hospital for management of acute physical health issues. Due to high opioid requirements and history of patient-initiated discharge, intravenous fentanyl was administered for treatment of opioid withdrawal, and management of pain, which supported continued hospitalization for acute care treatment and aligned with substance use treatment goals.

**Conclusion:**

This case demonstrates that intravenous fentanyl for management of OUD in hospital can be a feasible approach to meet opioid requirements and avoid fentanyl withdrawal among patients with severe OUD and daily fentanyl use, thereby promoting adherence to medical treatment and reducing the risk of patient-initiated discharge. There is an urgent need to tailor current treatment strategies for individuals who primarily use fentanyl. Carefully designed research is needed to further explore the use of IV fentanyl for acute care management of severe opioid withdrawal in a hospital setting.

## Background

The overdose crisis in North America is worsening, mainly driven by rapid changes in the drug markets, shifting towards increasingly potent synthetic opioids, primarily fentanyl and fentanyl analogues [1–3]. Compared to heroin, fentanyl is more potent, rapid acting, and shorter lasting [4]. Although studies across Canada and the US suggest that the majority of people who use drugs (PWUD) were unknowingly exposed to fentanyl through adulteration of street drugs in the past few years, recent evidence shows that more people are now using it knowingly and preferentially [5–8]. PWUD who intentionally seek out fentanyl report its strength as a primary motivator, and fentanyl preference has been associated with frequency of opioid use and overdose history [6, 7].

This creates unique challenges in the treatment and management of opioid use disorder (OUD) and its concurrent disorders [9–11]. For instance, appropriate management of opioid withdrawal and initiation of medication for OUD (MOUD) among hospitalized patients has been found to reduce risk of patient-initiated discharge and improve engagement in post-discharge OUD treatment [12, 13]. However, patients who use fentanyl have reported a higher likelihood of withdrawal and more difficult experiences with induction compared to those who use heroin or prescription opioids [14]. The rates of patient-initiated discharge and related complications, including incomplete treatment, worsening infection, and unintentional overdose, are already high among patients with OUD [15]. As well, PWUD often report distrust and anxiety when seeking care in the future as a result of insufficient and unpredictable pain and withdrawal management [16]. These outcomes are even more prominent among patients who primarily use fentanyl, especially as the pharmacological management of fentanyl withdrawal has so far mostly been identical to traditional opioid withdrawal approaches [17]. Treatment protocols in a hospital setting can therefore adapt to better address the needs of patients who use fentanyl, given the increased opioid requirements experienced by this patient population [18]. Exploring the use of high-potency opioids such as fentanyl in withdrawal management and treatment for severe OUD is an immediate priority and calls for significant clinical research efforts [10, 19–21].

We present a patient who was admitted to hospital for treatment of acute physical health complications relating to severe long-standing OUD and daily fentanyl injection, who received intravenous (IV) fentanyl in hospital to meet opioid requirements and avoid severe fentanyl withdrawal, thereby retaining the patient in care, improving treatment trajectory, and reducing risk of complications and overdose. We want to demonstrate the effective use of IV fentanyl for patients admitted to medical wards in order to support engagement in acute treatment as part of the clinical trajectory. We want to share this novel approach involving IV fentanyl to document the need of innovative clinical approaches in response to a crisis and underline the need for more clinical research in an emerging field. Written informed consent from the patient was obtained for participation and for publication of the case-report.

## Case presentation

A 42-year-old female with a long history of injection opioid and crystal methamphetamine use was admitted to acute care for bilateral lower limb cellulitis. She was started on IV antibiotics for the cellulitis, and referred to the Complex Pain and Addiction Service for assessment and management of her pain and substance use. The patient’s past psychiatric history includes severe opioid and stimulant use disorder, currently using IV fentanyl and IV crystal methamphetamine. Her urine drug screen on admission was positive for fentanyl, opiates, and methamphetamine. She had a history of Attention Deficit and Hyperactivity Disorder, but no other psychiatric history was documented. Her past medical history includes Methicillin-resistant Staphylococcus aureus (MRSA) bacteremia, septic arthritis, prolonged QT, type 2 diabetes, erosive esophagitis, hypertension, iron deficiency, and anemia.

The patient’s OUD was treated in the community with 200 mg IV diacetylmorphine (DAM) twice a day as part of the injectable opioid agonist treatment (iOAT) program as well as with 12-h extended-release morphine (M-Eslon) 200 mg twice a day. This treatment regimen is in accordance with provincial clinical guidance for iOAT and has been described in other recent case reports among people who use fentanyl [22–25]. Several randomized trials (such as the North American Opiate Medication Initiative (NAOMI) [26] and the Study to Assess Longer-term Opioid Medication Effectiveness (SALOME) [27]) have shown that iOAT is feasible, safe, and effective when treating individuals with long-term chronic injection opioid use and for whom the available medications for OUD have not been effective [28–30]. In addition, flexible doses of oral OAT for management of OUD are typically provided to patients receiving iOAT, as long-acting opioid such as SROM can help to reduce iOAT dose, frequency of daily injections, and reduce the severity of withdrawal symptoms by providing background 24-h opioid receptor saturation [22, 23]. In addition to her MOUD, she was injecting approximately 0.25 g of street fentanyl 3–4 times per day. In the three days leading to admission, she increased her dose to approximately 0.75 g due to lower extremity pain secondary to her cellulitis that prevented her from attending her iOAT appointments. When asked about her fentanyl use despite receipt of DAM, she described a preference for fentanyl due its superior effect relative to other opioids with regards to resolution of withdrawal, pain management, sedation, and intoxication. Historically, she has experienced difficulty meeting her opioid requirements on the medical unit and has a history of self-initiating premature discharge for this reason.

On admission, the patient was initially switched to IV hydromorphone at an equivalent dose to iOAT treatment. She declined further doses of hydromorphone relating to increased anxiety stating, “[hydromorphone] feels like a bad trip…it makes me agitated”. She also declined IV morphine options due to a history of significant pruritus. Moreover, DAM is not available in acute care settings in Canada. She therefore requested to be started on IV fentanyl, which she had been treated with during a previous admission five months prior and tolerated well. She expressed a plan to continue using illicit fentanyl during her admission or to self-initiate discharge if IV fentanyl was not provided. Although other treatment options (e.g., ultra-rapid buprenorphine micro-induction [31], iOAT and OAT adjustments) were considered and explored, her immediate goals were to remain on the existing community treatment program and continue her illicit fentanyl use at discharge.

For these reasons and based on medical indication for continued treatment with IV antibiotics, the patient was initiated on the IV fentanyl protocol with the purpose of meeting opioid requirements and maintaining admission to complete IV antibiotic regimen. (Table 1). Her initial loading dose was 800 mcg IV fentanyl, completed in 200 mcg pushes at 5-min intervals with a Clinical Opiate Withdrawal Score (COWS) dropping from 17 pre-induction to 2 post-induction (Table 2). She was then maintained on a dose of 400 mcg (equivalent to half of her induction dose) via symptom-suppressed protocol, determined by patient self-reported comfort and Richmond Agitation-Sedation Scale (RASS) between 0 and − 1 [32]. Although the protocol was intended for doses every hour (Table 1), a natural consolidation process took place whereby the 400 mcg IV fentanyl dose provided adequate sedation, pain relief and opioid effect and only requiring dosing every 3–4 h (Table 2).

**Table 1:** Symptom-triggered IV fentanyl induction orders

Phases	Medication	Monitoring
Pre-induction	Discontinue all opioids	
Induction	Fentanyl 100–200 mcg IV q5min until patient satisfaction and RASS 0/−1	COWS before/after induction RASS, vitals* after each dose
Maintenance (0–24 h post-induction)	Fentanyl X mcg IV q1h PRN to maintain patient comfort and RASS 0/−1, where X is 50% of cumulative induction dose	RASS, vitals* q1h Continuous ECG and oxygen saturation monitoring
Consolidation (24 h + post-induction)	Reduced frequency of dosing on consecutive days to q2h, q3h, q4h PRN, where fentanyl dose is calculated using 24 h cumulative dose divided by dosing frequency	RASS, vitals* q1h Continuous ECG and oxygen saturation monitoring
Oversedation (RASS ≤ -2)	Naloxone 0.1 mg IV push q2min PRN until patient awakens	RASS, vitals* q1h Continuous ECG and oxygen saturation monitoring

q_mins: every_minutes; q_h: every_hour; prn: as needed; IV: intravenous; RASS: Richmond Agitation-Sedation Scale; COWS: Clinical Opiate Withdrawal Score; mg: milligram; mcg: microgram

^*^Vitals: heart rate, blood pressure, respiratory rate, oxygen saturation

**Table 2:** Dosage regimen

Admission Timeline	Hydromorphone (IV)	Fentanyl (IV)			Monitoring
	Doses	Order	Dose	Cumulative daily	RASS (COWS)
Day 1	100 mg refused				
	100 mg				
	100 mg				
Day 2	100 mg refused				
	100 mg refused				
		100–200 mcg q5 min prn (induction)	200 mcg		0 (17)
			200 mcg		0
			200 mcg		0
			200 mcg	800 mcg	0 (2)
Day 3		300–400 mcg q1h prn (maintenance; consolidation)*	5 × 400 mcg	2000 mcg	− 1/0
Day 4			4 × 400 mcg; 1 × 300 mcg	1900 mcg	− 1/0
Day 5			3 × 400 mcg; 4 × 300 mcg	2400 mcg	− 1/0
Day 6			6 × 400 mcg	2400 mcg	− 1/0
Day 7			7 × 400 mcg	2800 mcg	− 1/0
Day 8			4 × 400 mcg	1600 mcg	− 1/0

q_mins: every_minutes; q_h: every_hour; prn: as needed; IV: intravenous; RASS: Richmond Agitation-Sedation Scale; COWS: Clinical Opiate Withdrawal Score; mg: milligram; mcg: microgram

^*^Patient self-selected for 300–400 mcg IV fentanyl every 3–4 h as it provided adequate sedation, pain relief and opioid effect

The patient remained in hospital for the duration of her IV antibiotic treatment and was transitioned to PO antibiotics. As she was no longer required to be in the hospital, she then continued her treatment in the community with resumption of her M-Eslon and iOAT programs. Although the IV fentanyl protocol was meeting her opioid requirements, she reported having quite significant cravings for methamphetamines.

## Discussion

The use of fentanyl as drug of choice is an emerging shift in North America. In an acute care setting for patients with severe OUD and daily injection fentanyl use, new treatment approaches need to be developed to avoid self-initiated discharge, overdose, spread of COVID-19, etc. [24].

In general, traditional approaches to opioid withdrawal have been used for fentanyl withdrawal management, but standard protocols have historically been unsuccessful at retaining patients in care due to severe opioid withdrawal, as demonstrated by frequent patient-initiated discharge, and drug recurrence [9, 18, 33]. This case is representative, as the patient resorted to using illicit fentanyl during admission and planned to self-initiate discharge to self-manage withdrawal symptoms, pain and cravings, which would have increased risk of sepsis, infection progression and other medical complications. Moreover, conventional opioids such as hydromorphone or morphine were not addressing the patient’s opioid requirements or were declined due to the patient's history of adverse experiences. Similarly, novel approaches such as ultra-rapid or rapid buprenorphine micro-induction, or transdermal fentanyl bridging did not align with the patient's goals and preferences of remaining on iOAT (IV diacetylmorphine) and OAT (M-Elson) programs [31, 34–36]. Indeed, the patient identified her existing outpatient treatment as helpful, effective in reducing withdrawal, cravings, and use of illicit substances, and did not express interest in transitioning from this. Therefore, in this situation, providing fentanyl, a pharmacologic agent that the patient was accustomed to, was an effective approach to support patient engagement in acute treatment and appropriate management of presenting illness.

Improving outcomes among patients with OUD occurs through patient-centered care built on a mutually respectful, shared decision-making, and an understanding therapeutic relationship, which requires time and space [37]. For the patient in this case report, unmet opioid requirements and severe opioid withdrawal created a hostile environment that was unfavourable to treatment, often leading to premature patient-initiated discharge and adverse outcomes, along with further patient distrust of the healthcare system. Administration of IV fentanyl allowed for an enhanced therapeutic relationship and individualized care plan addressing the needs and preferences of the patient, while also retaining her in care. Furthermore, the appropriate management of fentanyl withdrawal with IV fentanyl eliminated potential safety concerns from healthcare staff and other patients, who can be the target of verbal and physical aggression and possible assault in the setting of severe opioid withdrawal [38]. In the context of the COVID-19 pandemic, protocols which increase treatment completion and reduce self-initiated discharge, such as acute use of IV fentanyl for OUD management, also reduce hospital re-admission thereby limiting potential infection spread and ameliorating the safety of the community [24].

This is a pragmatic approach which focuses on immediate patient needs, patient-chosen goals, and reducing the harms experienced by individuals who are at an extraordinarily high-risk of mortality and morbidity. It was effective to maintain a hospitalized patient with daily injection fentanyl use on their OAT trajectory and support them in achieving their therapeutic goals, which was to continue her iOAT and M-Eslon treatment programs without complications or set-backs.

There is a dearth of research addressing the unique challenges of primary fentanyl use and a lack of studies evaluating the effectiveness of current standard protocols and treatment options among fentanyl-using patients [39]. For instance, although retention and abstinence outcomes for buprenorphine have been shown to be similar between patients with primary fentanyl use relative to primary heroin use, fentanyl exposure has also been independently associated with OAT dissatisfaction [18, 40]. This highlights the urgent need to tailor current treatment strategies for individuals who primarily use fentanyl, given the widespread prevalence and ubiquity of fentanyl in North America. Public health efforts should adapt to evolving drug market trends and patterns of use in order to provide better health outcomes among PWUD and decrease overdose fatalities. More generally, the concept of using prescribed fentanyl as MOUD ("fentanyl-assisted treatment") has been gaining traction among clinical and academic forums [20]. This is analogous to using diacetylmorphine ("heroin-assisted treatment") which is currently regulated in several European countries and Canada, and has been proven more clinically effective and cost-effective than oral methadone for individuals with treatment-refractory OUD [41, 42]. Moreover, though fentanyl patches are being explored for treatment as MOUD in Vancouver, this is largely experimental and there is relatively limited basis for this currently [19]. Large scale clinical research efforts are urgently needed, while also considering the medical and legal implications of such interventions.

## Conclusion

Within the healthcare system, and especially in an inpatient setting, individuals who use drugs often experience insufficient pain and withdrawal management which results in distrust, illicit substance use of during admission, decisions to self-initiate discharge, and anxiety when seeking care in the future. These outcomes are even more prominent among patients who primarily use fentanyl. Given changes in drug markets and patterns of use, developing explorative treatment approaches for severe OUD in the age of fentanyl represents an immediate priority. The present case describes the feasibility of using IV fentanyl among individuals with severe OUD and daily intentional use of fentanyl in order to meet opioid requirements and to avoid fentanyl withdrawal, thereby reducing risk of patient-initiated discharge, improving retention in care and promoting adherence to the medical treatment plan. It documents a clinical response to a critical situation and calls for more necessary research efforts during this ongoing and worsening overdose crisis.

## Data Availability

Data sharing is not applicable to this article as no datasets were generated or analysed during the current study.

## Abbreviations

PWUD: People who use drugs
OUD: Opioid use disorder
IV: Intravenous
VGH: Vancouver general hospital
DAM: Diacetylmorphine
iOAT: Injectable opioid agonist treatment
OAT: Opioid agonist treatment
COWS: Clinical Opiate Withdrawal Score
RASS: Richmond Agitation-Sedation Scale
MOUD: Medication for opioid use disorder
